# Energy drinks consumption and perceptions among University Students in Beirut, Lebanon: A mixed methods approach

**DOI:** 10.1371/journal.pone.0232199

**Published:** 2020-04-30

**Authors:** Malake Ghozayel, Ali Ghaddar, Ghada Farhat, Lara Nasreddine, Janine Kara, Lamis Jomaa

**Affiliations:** 1 Department of Nutrition and Food Sciences, Faculty of Agricultural and Food Sciences, American University of Beirut, Beirut, Lebanon; 2 Department of Biomedical Sciences, Lebanese International University, Beirut, Lebanon; 3 Hubert Department of Global Health, Rollins School of Public Health, Emory University, Atlanta, Georgia, United States of America; 4 Social and Behavioral Sciences Department, Yale School of Public Health, Yale University, New Haven, Connecticut, United States of America; Northumbria University, UNITED KINGDOM

## Abstract

**Background:**

Energy drinks (ED) are caffeine- and sugar-rich beverages with other ingredients that are marketed for their energy-boosting and performance-enhancing effects. The consumption of these drinks, with and without alcohol, is dramatically increasing worldwide, despite the reported side effects and potential harms to consumers. Few studies, to date, have explored the perceptions and experiences of young adults towards these beverages.

**Objective:**

The present study aimed to explore the consumption patterns and correlates of ED consumption, as well as the perceptions and experiences of university students in Lebanon towards these beverages.

**Methods:**

A sequential explanatory mixed-methods approach was adopted. Data collection was conducted in two private universities in Beirut, Lebanon. A self-administered 36-item quantitative survey was first used to explore the prevalence and correlates of ED consumption among a convenience sample of university students (n = 226). The survey included questions related to socio-demographic characteristics, anthropometric measurements, and other lifestyle behaviors, including physical activity of university students. The subsequent qualitative phase consisted of focus group discussions (FGD) conducted to further examine the perceptions and experiences of university students towards ED. Descriptive statistics and logistic regression analyses were conducted using survey data, whereas the transcribed FGD were analyzed thematically.

**Results:**

A total of 226 university students completed the survey. Results showed that 45% of survey respondents consumed ED at least once in their life (*ever consumers*), among which 30% reported consumption of ED mixed with alcohol (AmED). Adjusting for socio-demographic and anthropometric characteristics, coffee and sports drinks consumption were significantly associated with ED (OR = 2.45, 95% CI = 1.20, 5.00, and OR = 4.88, 95%CI = 2.41, 9.88, respectively). In addition, physically active participants were 1.89 times (95%CI = 1.01, 3.51) more likely to consume ED compared to their inactive counterparts. During the qualitative phase, a total of six FGD were conducted with 29 university students, who reported consuming ED at least once per month. Three main themes were derived reflecting individual-, interpersonal/social- and environmental-level factors affecting ED consumption among university students. These themes were further supported by eight subthemes, including: perceived benefits of ED, experienced side effects, misinformation about content of ED, peer pressure and social image, as well as affordability and accessibility of ED.

**Conclusion:**

Findings highlight the need for public health policies and programs to curb the growing public health phenomenon of ED consumption amongst university students. Such programs should consider the multi-level factors affecting ED consumption at the individual, interpersonal/social, and environmental levels, including educational campaigns on ED potential harms, regulating ED content and labeling, as well as restricting sales and marketing of these beverages, especially among young consumers.

## Introduction

Energy drinks (ED) represent a wide range of beverages that usually contain high amounts of caffeine and sugar, in addition to ingredients, such as amino acids and herbal extracts [1, 2]. Although there is no universally agreed definition of ED, the market and popularity of these beverages have been increasing dramatically over the past two decades, and their consumption has become a widespread phenomenon [3]. In fact, the popularity of ED has been mostly witnessed among young adults, whereby more than 50% of the global consumer market consists of adolescents and young adults under the age of 35 years [4].

The popularity of ED among young adults could be attributed to their extensive promotion as products that increase energy, enhance mental alertness, and improve physical performance [5]. In fact, studies show that the most commonly reported benefits of ED include enhancing physical and mental performance [4, 6], improving alertness and concentration levels [7], and promoting the feeling of pleasure [8]. Although evidence does exist to support some of these effects [9], most of the reported benefits of ED are attributed to their caffeine content. Other common ingredients of ED include B-vitamins, derivatives of amino acids such as taurine, carnitine, creatine, and herbal extracts like guarana, ginseng, ginkgo biloba, which can contribute to the increased feelings of strength and stamina among consumers. Over the last few decades, the trend of ED mixed with alcohol (AmED) gained increasing popularity, especially among adolescents and young adults, due to the aggressive marketing of these beverages together with the appeal and anticipated benefits to consumers [10, 11]. Yet, major questions were raised, in parallel, regarding the safety of these beverages and potential harms from ED and AmED consumption [12, 13].

A growing body of scientific evidence shows that ED and AmED can have serious short and long-term health effects, particularly among children, adolescents, and young adults [14, 15]. The most frequently reported side effects of ED among consumers to date include tachycardia [8, 11, 16], insomnia [16], headaches [7, 8], frequent urination, nausea, vomiting, nervousness, abdominal pain, dyspnea, hypotension, dizziness, and redness of the skin [8]. Other studies highlight a number of adverse social, emotional, and behavioral outcomes reported by consumers of ED, whether consumed alone or mixed with alcohol. These side effects include daytime sleepiness and anger [17], irritability, and restlessness [18], increased risk of “getting into trouble at home, school, or work”, and being involved in violent behaviors and conduct disorders [13, 17].

Studies have shown that the prevalence of consuming caffeinated ED and AmED has been growing remarkably over the years, particularly among youth and young adults. In the United States [19], ED consumption was reported to reach 51% of college students, which was consistent with consumption patterns reported in other developed nations including Australia [20], Canada [21], and Italy [6]. In parallel, there has been a growing interest to assess the prevalence of this drinking behavior amongst young adults enrolled in colleges within several countries in the Middle East and North Africa (MENA) region. Findings from MENA countries showed that the rates of ED consumption varied between 50% to 64% in Kingdom of Saudi Arabia (KSA) [1, 22], Iraq [23], and Lebanon [8], with the prevalence of this drinking behavior reaching as high as 92% of college students in the United Arab Emirates (UAE) [24]. The high rates of ED consumption reported in MENA countries raise serious concerns as to the potential implications of this drinking behavior on the health and safety of young adults [24, 25].

Despite the worldwide popularity of ED, to date, few studies explored qualitatively the perceptions and experiences of adolescents and young adults towards these caffeinated beverages. Such qualitative investigations were conducted mainly in Australia and New Zealand [12, 26, 27]. One of which used a mixed-method approach to investigate adolescents' perceptions of AmED and the factors associated with their use [12]. Results from these studies, and a review conducted by Visram and colleagues on the patterns and attitudes of children and young people towards ED, highlighted the role of branding and marketing as the main influences on the use of these beverages. In addition, consumers sought these beverages for their taste and stimulating effects with minimal awareness of their negative health effects [14]. Yet, with the rising prevalence of ED consumption in both high- [19–21] and low-to-middle-income countries (LMICs) [11, 28], it is important to further understand the perceptions and attitudes of young consumers towards these beverages and explore the facilitators and barriers for their drinking behaviors. The present study aims to explore the consumption patterns and correlates of ED and AmED consumption among university students in Lebanon, as well as the perceptions and experiences towards these beverages. The study findings would provide the evidence-base needed to devise public health policies and programs to limit the consumption of ED and AmED as well as their potential harms on consumers, particularly young adults.

## Methods

A sequential explanatory mixed methods approach was adopted in the present study. The first phase included a quantitative survey to explore the prevalence and correlates of ED consumption among university students in Lebanon. The second phase aimed at examining the perceptions and experiences of university students towards ED using focus group discussions. The two phases of the study were conducted among university students recruited from two private universities in Beirut, the capital of Lebanon. The two universities were selected to represent students from different socioeconomic backgrounds within an urban setting, taking into consideration that the credit cost in one university is four times higher than that of the other university.

### Ethical considerations

The study was conducted in accordance with the Declaration of Helsinki, and approved by the Institutional Review Boards of the two academic institutions involved in the study. Informed consent was secured from all study participants for both phases of the present study prior to data collection. In addition, participants of the focus groups were requested to provide their written consent for audio recording the discussion and for anonymously quoting any parts of the interview in published materials. Confidentiality of information collected throughout the study phases and the privacy of participants were ensured by the research team. Participation in both phases of the study was completely on a voluntary basis and university students had the right to refuse to participate or withdraw from the study at any time without any penalties or repercussions.

### Quantitative survey *(first phase)*

#### Recruitment and data collection

During the academic year 2013–2014, a convenience sample of 240 university students was recruited to participate in the first phase of the project. A priori sample size calculations showed that 225 participants were needed to provide a 95% confidence interval around a 50% prevalence estimate for ED consumption with a precision of ±6.5%. The prevalence estimate (50%) used in the sample size calculations was based on previous investigations of ‘*ever*’ consuming ED (consumption of ED at least once in a lifetime) in Lebanon and other similar settings in the MENA region [8, 11].

Students were directly approached for participation on their respective campuses and were verbally screened for eligibility by trained field surveyors. To participate in the survey, students had to be Lebanese, 18 years of age or older, and currently enrolled in one of the two private universities included in the study. Upon securing their written consent, interested and eligible university students were asked to complete a self-administered questionnaire (paper-version) with the assistance of the trained surveyors. A total of 36 closed-ended questions were included in the survey covering information related to consumption of ED and other selected beverages (including alcohol, sports drinks, sodas, and coffee), socio-demographic characteristics, anthropometric measurements, and physical activity. Socio-demographic questions included participant’s age, gender, household (parental) monthly income (<1500, 1500–2000, and >2000 US dollars), educational level (undergraduate, graduate), and field of study (health-related major, such as biology, chemistry, nursing, nutrition, or non-health related major). Anthropometric data were self-reported and included weight in kilograms (kg) and height in meters (m). The body mass index (BMI) was subsequently calculated as weight divided by height squared (kg/m^2^), and BMI status was identified based on the Center for Disease Control and Prevention classification for young adults: individuals with BMI between 18.5 and 24.9 kg/m^2^ were categorized as “normal body weight”, and those with BMI ≥ 25 kg/m^2^ were classified as overweight. Questions reporting frequency of beverages consumption, including ED and AmED, were adapted from the Harvard Food Frequency Questionnaire [29]. The physical activity of survey respondents was assessed by deriving Metabolic Equivalent of Tasks (METs) from the frequency (times per week), duration (minutes per time), and intensity of the performed exercise. METs are physiological measures expressing the cost of physical activities and were calculated in accordance with the standard IPAQ scoring procedures [30]. Survey respondents were then classified into one of the following groups: "low" physical activity (< 500 MET/min/week), "moderate" physical activity (500–2500 MET/min/week), or "vigorous" physical activity (> 2500 MET/min/week). Respondents involved in moderate and vigorous activities were later categorized as ‘physically active’, while those of low physical activity were categorized as ‘physically inactive’ [31].

Questions related to ED included preferred brands, places of purchase, times of consumption, and consumption patterns, such as mixing with other beverages (mainly alcohol). In line with similar studies investigating ED consumption [8, 22, 25], survey respondents were asked if they *ever* consumed ED (Yes or No). In addition, the frequency of ED consumption was reported in the fall/spring (October till May) and summer (June to September) semesters, from which we derived the number of *regular* ED consumers (drink at least 1 ED can per month) [19]. Moreover, survey respondents were asked to rate (on a scale from 1 to 5) the importance of a list of reasons for the consumption of ED, including drinking because their close friends drink it, because their siblings drink it, because it is cheap, to get energy to study, to work or to play. Respondents were also asked about the frequency of experiencing symptoms and side effects after consuming ED and/or AmED, including headaches, nausea, vomiting, fatigue, as well as the frequency of engaging in risky behaviors such as getting into an argument or fight, not wearing a seatbelt, and not using a condom in sexual practices. The compiled questionnaire was pilot-tested among 20 university students (10 per university) to ensure cultural appropriateness and clarity prior to data collection.

#### Data analysis

Continuous variables were presented as means and standard deviation (SD), whereas categorical variables were reported as counts and percentages. Independent sample t-tests were used to determine differences between *ever* ED consumers and non-consumers with respect to age (years), BMI (kg/m^2^), and physical activity (metabolic equivalents/min/week). Chi-square analyses were used to examine associations between consumption of ED and categorical variables, including gender, BMI status (normal *vs* overweight), physical activity level (active *vs* inactive), and consumption of selected caffeinated beverages. Logistic regression analysis was conducted to explore socio-demographic, anthropometric, and lifestyle characteristics of university students (including age, gender, income, field of study, education level, physical activity level, and consumption of selected beverages) as correlates of ED consumption. Data analysis was performed using SPSS software (version 20.0). A p-value <0.05 was considered statistically significant.

### Qualitative focus group discussions *(second phase)*

#### Recruitment and data collection

To further explore the perceptions and attitudes of regular ED consumers, focus group discussions (FGD) were conducted with a convenience sample of students enrolled in one of the two private universities included in the first phase of the study. Data collection was carried out during the spring and early summer of 2014–2015 academic year. Inclusion criteria for students to be recruited for the FGD included the following: 1) Lebanese nationality, 2) age between 18 and 30 years, and 3) regular consumption of ED, defined as consuming at least 1 ED per month [19]. FGD participants were approached face-to-face by one of the research team members (MG) in both university campuses, and those who met the inclusion criteria were invited to participate in the FGD. In addition, flyers were posted around both campuses for those who would be interested to take part in the study. Interested students were assigned to one FGD according to gender and university location and were gathered in safe, private, and quiet meeting rooms within their respective campuses. Segregating focus groups by gender was deemed important within the local context, whereby some of the drinking and lifestyle behaviors that were discussed as part of the FGD could be perceived as socially unfavorable or unacceptable. For instance, ED consumption was associated with alcohol drinking and cigarette smoking [32–34], both of which are perceived as inappropriate behaviors in conservative settings [35]. All FGD participants signed written consent forms prior to data collection and their approval was secured to audio-record the discussion.

Prior to the start of each FGD, students were also given a self-administered brief questionnaire (paper-version) to complete. Questions included age, gender, educational level, marital status, household income, as well as questions about ED and alcohol consumption, anthropometric characteristics, and lifestyle behaviors (including physical activity and sleeping patterns). One team member (MG) served as the moderator for all the FGD and sat in the center of the U-shaped seated group of participants to facilitate the free flow of interaction and communication with participants, while another team member (SJ) acted as an observer and note taker. MG is a female graduate student who received training on responsible and ethical conduct of research as part of her studies and received refresher training on how to moderate FGD prior to data collection. MG introduced herself and briefly introduced the purpose of her project and the observer (SJ) who joined her during the FGD. The observer sat in the back of the arranged circular setting to observe and take notes. A pre-assigned code that uniquely identified each respondent was used during the note-taking process where verbal and non-verbal responses and reactions were documented. Selected ED brands commonly available in the local market were placed in the room as visual stimuli to ensure all participants were aware of the beverages being discussed and to trigger any thoughts or memories related to these beverages. The moderator followed a semi-structured focus group script that was developed by the study investigators based on existing literature and preliminary findings from the quantitative survey. The focus group script included open-ended questions with probing queries to help stimulate the discussion and clarify any ambiguities. Key questions covered various areas of the researched subject including first experience with ED and/or AmED, reasons for consumption, opinions of peers and parents towards these beverages, and potential facilitators and barriers to consumption (S1 Appendix).

Each FGD included an average of six participants. FGD were conducted in colloquial Arabic and lasted 45–60 minutes on average. These FGD were audio-recorded, transcribed verbatim in colloquial Arabic, and then were translated to English by the moderator (MG). The translations were further reviewed by LJ and any discrepancies were corrected, as needed.

#### Data analysis

After transcription of FGD, the moderator (MG) coded participants’ quotes and presented them, as such, focus group discussion 1, male participant 1 (FG1M1). Transcripts were compared to notes taken during the FGD to capture verbal and nonverbal messages delivered by the participants. The researchers (MG & LJ) conducted content analysis to identify the main categories and subcategories extrapolated from the transcripts by identifying implicit and explicit meanings to common ideas shared by participants. Content analysis approaches to qualitative data are highly reliable and valid even though there is no strict set of steps for content analysis, and typically different approaches are regularly utilized and accepted in public health research [36]. Identified ideas were then coded and refined into three main themes and eight different subthemes. After further readings and discussions, all research team members (authors) compared and contrasted coded data and merged the identified subthemes into three final themes matching the socio-ecological framework. A description for each theme and subtheme was written and illustrated by quotes extracted from the transcripts, as detailed in the results section.

The research team ensured that credibility and reflexivity were observed to increase the rigor of the data collection and analysis. In terms of credibility, the research team relied on the transcribed, audio-recorded FGD as the main data repository supported by mental notes and the observers’ notes. In addition, themes and subthemes were supported with quotes and every attempt was made to maintain a level of reflexivity at each stage of the research. Reflexivity refers to self-examination implemented by the moderator, which includes keeping mental and written notes after the FGD to identify whether participants’ viewpoints were influenced by the researcher [37]. During data analysis, frequent team meetings were conducted to compare and contrast emerging themes while putting priority on the collected data backed up by quotes from participants. In addition, the research team followed the COnsolidated criteria for REporting Qualitative research (COREQ) checklist for reporting this study (see S2 Appendix).

## Results and discussion

### Descriptive characteristics of the study participants

For the quantitative survey, a total of 226 respondents had complete data and were included in the final analysis of the paper. The average age of survey respondents was 20.7 (SD = 0.13), and 53.5% were females. The majority of respondents (93%) were undergraduate students, and 35% were enrolled in a health-related field of study. In addition, 45% of the survey respondents consumed EDs at least once in their lifetime (*ever consumers*), of which 53.8% reported drinking at least one can per month (defined as *regular consumption)*. In addition, 30% of survey respondents reported trying or drinking AmED at least once in their lifetime. Socio-demographic, anthropometric, and lifestyle characteristics of survey respondents by ED consumption were presented in Table 1. For the qualitative phase, six FGD were conducted after which data saturation was reached. A total of 29 university students, who reported consuming ED at least once per month, participated in the FGD.

**Table 1:** Baseline characteristics of survey respondents, by energy drinks (ED) consumption (n = 226).

	Total	ED ever consumers	ED non-consumers	*p-value*†
	(n = 226)	(n = 102)	(n = 124)	
***Demographic characteristics***				
**Age (years)**	20.67±0.13	20.62±0.18	20.72±0.18	0.707
**Gender**				**0.004**
Male	105 (46.5)	58 (56.9)	47 (37.9)	
Female	121 (53.5)	44 (43.1)	77 (62.1)	
**Monthly household income (USD)**				
<1500	76 (36.9)	32 (32.7)	44 (40.7)	0.483
1500–2000	31 (15.0)	16 (16.3)	15 (13.9)	
>2000	99 (48.1)	50 (51)	49 (45.4)	
**Education**				0.924
Undergraduate	209 (92.9)	94 (93.1)	115 (92.7)	
Graduate	16 (7.1)	7 (6.9)	9 (7.3)	
**Health-related field of study**	79 (35.4)	36 (36.4)	43 (34.7)	0.794
***Anthropometric characteristics***				
**BMI(kg/m**^**2**^**)**	23.34 ± 0.25	23.60±0.39	23.12±0.31	0.338
**BMI status**				
Normal weight (18.5–24.9kg/m^2^)	154 (70.6)	65 (66.3)	89 (74.2)	0.206
Overweight (≥ 25 kg/m2)	64 (29.4)	33 (33.7)	31 (25.8)	
***Physical Activity***				
**METs/min/week**	1117.57±99.16	1230.51±144.97	997.83±135.79	0.243
**PA status**‡				**0.001**
Inactive	106 (47.7)	35 (35.4)	71(58.2)	
Active	116 (52.3)	64 (64.6)	51(41.8)	
***Ever consumption of selected beverages***				
**Alcohol (Yes)**	78 (39.4)	44 (47.3)	34 (32.4)	**0.032**
**Coffee (Yes)**	168 (74.3)	85 (83.3)	83 (66.9)	**0.005**
**Sports drinks (Yes)**	67 (29.6)	49 (48.0)	18 (14.5)	**<0.001**
**Sodas (Yes)**	193 (85.4)	89 (87.3)	104 (83.9)	0.473

† p-values were derived based on independent t-tests for categorical variables and chi-square analyses for categorical variables.

‡Participants were categorized as active, if involved in regular moderate and vigorous activities (such as brisk walking, jogging, aerobics, dancing), and as inactive, if they reported no activity or being involved in light activities (including stretching, yoga, slow walking).

### Consumption patterns of energy drinks amongst survey respondents and focus group participants

Findings from our survey analysis showed that 45% of respondents tried ED at least once in their life, with more than half consuming it on a regular basis. These results were in accordance with those reported on ED consumption among young adults in several studies conducted in nearby Middle Eastern countries, including Turkey [11], Iraq [23], and the Kingdom of Saudi Arabia [22], with approximately half of surveyed college students reporting ever consuming ED. Nevertheless, the rate of ED consumption amongst university students in the current study was lower than those reported in two previous studies conducted in Lebanon, (64%) [8] and (77%) [38], and much lower than those reported in UAE (92%) [24]. The observed differences across studies could be attributed, in part, to how researchers identified ED consumers, based on *ever* or *current* consumption as well as the frequency of intake. Variations were also noted in terms of age groups included in these studies, as well as differences in the rigor of study designs and sampling sizes and methodologies adopted across studies. For example, ED consumption was assessed amongst school- and college-aged students (13–30 years old) by Itany *et al*. [8], whereas our study included only university students (age range: 18 to 30 years). In addition, Dwaidy and colleagues [38] used a small sample of university students from one university (n = 100) in Lebanon and 70% of participants were males, which may explain the high proportion of ED reported in their study sample compared to our results (77% and 45%, respectively).

Gender differences in the consumption behaviors and perceptions towards ED were, in fact, noted in both phases of the present study. Male participants in the quantitative survey were found to be significantly more likely to consume ED compared to females (57% vs. 43%, p = 0.004). These findings were later validated by the opinions of some male participants in the FGD within the present study, who highlighted that the consumption of ED by females is only “acceptable” when it is non-alcoholic. Male FGD participants further reported that the image of a young woman drinking AmED in public is not an attractive behavior and rather socially undesirable: *"Alcohol in public places*? *No*, *this is very inappropriate" (FG1M)*. The higher consumption of ED and alcoholic beverages among male college students when compared to females was also reported in previous studies [1, 22, 39]. A possible explanation for the higher intake and favorable perception towards ED among males is that they are the primary target of ED advertisements [34]. In fact, advertisements were noted to associate ED consumption with masculinity and with expectations of increased attraction to the opposite sex [40]. Another possible reason for the difference in consumption behaviors is the less social desirability of this drinking behavior, especially if mixed with alcohol, among females due to local cultural norms. These findings are further echoed by a recent study conducted in the United States exploring the perceptions towards women who drink in social contexts. Researchers found that women drinking alcohol in public spaces are more likely to be seen negatively, to be dehumanized and perceived to be more sexually available by both genders, whereas men who drink alcohol were not perceived as negatively [41].

#### Correlates of energy drinks consumption amongst survey respondents

When exploring correlates of ED consumption amongst survey respondents in the current study, results showed significant associations between ED consumption with being physically active (OR = 1.89, 95% CI: 1.01, 3.51) and with other beverages, including coffee and sports drinks (OR = 2.45, 95% CI = 1.20, 5.00 and OR = 4.88, 95%CI = 2.41, 9.88, respectively), even after adjusting for other socio-demographic correlates such as age and gender (Table 2).

**Table 2:** Correlates of energy drinks consumption among survey respondents (n = 169).

	Adjusted Odds Ratio^†^	95% Confidence Interval^†^
**Age (years)**	0.99	0.85, 1.15
**Gender** (reference = male)	0.79	0.42, 1.50
**Physical activity** (reference = inactive)	1.89	1.01, 3.51
**Coffee consumption**(reference = no)	2.45	1.20, 5.00
**Sports drink consumption** (reference = no)	4.88	2.40, 9.80

†Adjusted odds ratios (ORs) were presented with 95% confidence interval (CI) using multiple logistic regression analysis. The model was adjusted for socio-demographic characteristics (age, and gender), physical activity, coffee consumption and sports drink consumptions.

Results from our survey analysis were found to be in agreement with findings from other studies showing that ED consumption is a growing and common trend among college students, particularly athletes [16, 42]. Improving sports performance was similarly reported as major reason to consume ED by participants of studies conducted in Argentina [28] and Australia [27]. Positive effects, such as enhancing physical performance [4, 6], decreasing fatigue, increasing stamina, and improving overall physical capacity [43], are the main messages communicated to adolescents and young adults through ED advertisement campaigns [3, 34], which explains higher ED consumption among athletes. The strong association between ED and sports drinks may be attributed to the misperceptions of consumers that these beverages are alike and have similar hydrating properties [8, 39, 44]. There is also the possibility that consumers of ED do not necessarily differentiate between sports drinks and ED. In a study investigating the perceptions of US youth about ED consumption, it was noticed that participants got confused between both forms of beverages and considered ED and sports drinks as different versions of the same beverage [44]. Similarly, around 16% of surveyed school and university students in Lebanon considered ED as sports drinks, which may possibly explain the positive association between the consumption of these two beverages [8]. Given that both coffee and ED are also rich sources of caffeine, it is not surprising to observe a strong association between both types of beverages among university students. Scientific findings about the effect of ED on physical performance revealed that possible effects such as alertness, reaction time, and performance in aerobic and anaerobic activities are mainly caffeine dose-dependent [45]. However, it is worth noting that a review conducted by Visram and colleagues to examine patterns of ED use among children and young individuals showed that the highest ED consumption was not only observed among highly active individuals but also among those who had the most sedentary lifestyle [14].

### Factors affecting energy drinks consumption among focus group participants

**Fig 1:**
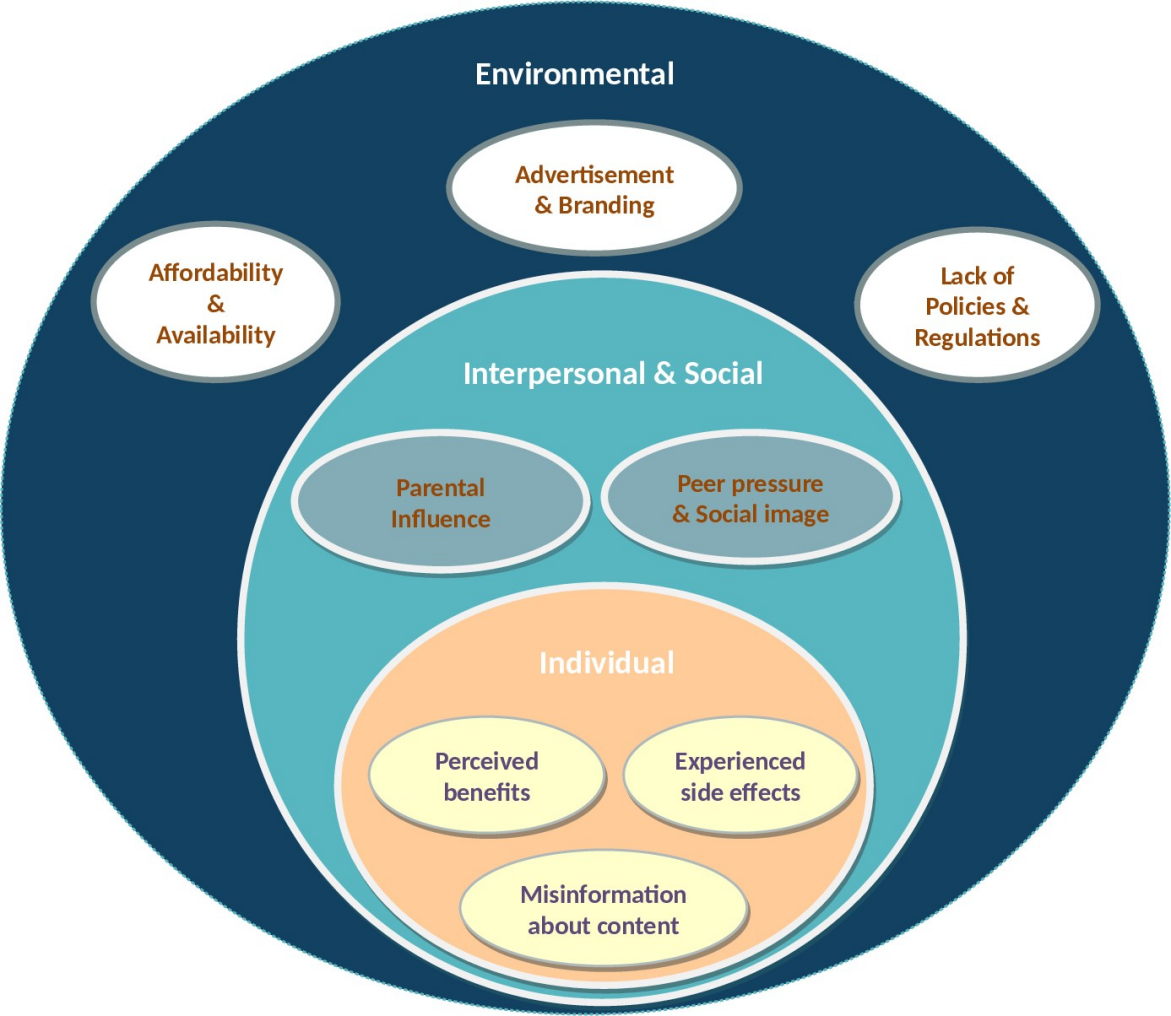
A socio-ecological framework including individual-, social- and environmental-level factors influencing energy drinks consumption by Lebanese university students.

When exploring the perceptions and experiences of university students towards ED consumption, three main themes emerged from the FGD, namely: individual-level factors, interpersonal factors, and environmental-level factors. These themes were further supported by eight subthemes, including perceived benefits from consumption, experienced side effects, parental influence, peer pressure, social image, and advertisement and branding (Fig 1).

The three main themes that emerged were in alignment with the social-ecological model (SEM) [46] showing that ED consumption can be influenced by a multi-level array of factors highlighting individual, interpersonal/social, and environmental aspects of the framework [47]. Previous studies assessing similar eating and drinking behaviors, including alcohol [48, 49] and soft drink use [50], have identified components of the SEM to affect consumer behaviors. The SEM has been also used in the scientific literature as a framework for designing interventions to increase the consumption of fruits and vegetables [51] or decrease the consumption of alcohol or sugar-sweetened beverages [49, 52]. Several themes and subthemes that emerged from FGD supported findings from the survey analysis.

A comparison of these findings from both phases of the study together with supportive quotes from participants is presented in Table 3. A detailed discussion of these results is presented below.

**Table 3:** Summary of themes and sub-themes emerging from focus group discussions (with supportive quotes) in comparison to results from survey analysis conducted among university students in our study.

Qualitative phase (focus group discussions)			Quantitative phase (survey results)
Themes	Sub-themes	Supportive quotes	n(%)*
**Individual-level factors**	1- Perceived benefits of ED (provides energy to study, workout, stay awake)	*"Honestly*, *ED are very important…to study for example*.*"* (FG1M9)	• Getting energy to study:47% • Getting energy to work:36% • Getting energy to play sports:35% • Staying awake/ preventing sleep: 20%
		*"We don't really drink ED but it's mostly during the final period or something*, *we need to stay awake*.*"* (FG5F1)	
		*“When I’m tired and want to play football or basketball*. *I drink ED and I feel I have more energy*.*”* (FG2M4)	
		*“I drink it to get energy when I have something to do or when I didn’t get enough sleep*, *it gives me a boost*.*”*(FG1M8)	
		*“When you need to stay awake you’ll take anything*.*”* (FG4F5)	
	2- Experienced side effects did not affect ED consumption(dizziness, shakiness, increased heart beat)	*“When I first started drinking ED I used to feel dizzy but now I got used to it”* (FG2M1)	• Increased heart beat:42% • Shakiness: 36% • Dizziness: 30% Anxiety/ nervousness/ irritability: 29%
		*“It happened once to me*, *I lost count of how many I was drinking*, *because it was new year and everybody was drinking*. *I couldn't sleep all night and my heart was beating fast*.*”* (FG5F3)	
		*“When I smoke shisha and drink ED I feel my heart beating fast*.*”*(FG4F5)	
		*“I heard that someone in the university drank ED after gym and died*.*”* (FG6F3)	
	3- Misinformation about content of EDs and lack of knowledge about risks	*"I heard it has cows’ or horses’ sperm but I didn’t believe it*!*"* (FG2M3)	-
		*"Red Bull is good…athletes can take it*, *because in its ingredients it has a high concentration of ions*, *so it's fine" (FG4F3)*	
		“*Main ingredients are sugar*, *caffeine and colourings maybe” (FG2M1)*	
**Interpersonal/social factors enabling ED consumption**	4- Peer pressure& social image *(Close friends drink AmED*, *drink when celebrating*, *socializing)*	*"My friends were all drinking the vodka ones [AmED] so I tried it"* (FG6F1)	• Close friends drink AmED: 15% • Drinking to celebrate: 47% Drinking to socialize: 33%
		*“If my friends and I are drinking alcohol and someone in the group doesn’t want to*, *I encourage him to get an ED instead of vodka or whiskey*.*”* (FG6F4)	
		*“As I grew up and we started hanging out and partying*, *the most common drink was alcohol mixed to energy drink it was either [ED brand] and vodka or vodka with another type of alcohol so from that point when all my friend were drinking this I'm not gonna be the only one to drink that*, *I used to go with the flow*. *It was like my friends encouraged me to have a sip” (*FG3M1).	
	5- Parental influence	*" They're not against it because they know when we're studying it's fine but if I'm gonna go drink it for fun I don’t think they'll be very happy about it""* (FG6F2)	
		*"I can't tell my parents that I drink [energy drink brand]*. *It's not that I'm hiding a secret but I'd rather not listen to lectures so I'm fine*, *when I want to drink I do it outside "*(FG4F7).	
		*" They even argue about soda*, *so ED to them is catastrophic but anyway"* (FG2M3)	
**Environmental-level factors promoting ED consumption**	6- Advertisement & branding	*"I'm influenced by the ads*, *most of the times I buy it just to get it*, *I only sip a little bit*" (FG4F)	-
		*“The ads attract me the most*. *I mostly like the one with the guy in the gym and all girls are looking at him” (FG2M1)*.	
		*“What I like is that they make you believe that you can do better just from drinking ED*. *I like extreme sports and everything related to it so when Red Bull used extreme sports in their ads they made me like them (encouraged me) more” (FG3M1)*	
	7- Affordability & availability	*"A lot of people won't admit buying the cheaper ED because of the price*. *But actually*, *it has the same taste*, *they would buy 2 for the price of 1*!*" (FG4F7)*	Easy access in gas stations:29% Easy access in supermarkets:24%
		*" I live in dorms so it's very close to me when I need it I easily find it and buy it" (FG5F2)*	
		*"I don't keep extra bottles*, *I just get them from any shop around" (FG3M1)*	
		*"ED prices are reasonable" (FG1M- several)*	
	8- Lack of policies &regulations (marketing, points of sale, and minimum age)	*"I didn't hear of any [policies]*. *Maybe they can start with the ones mixed with alcohol because we know this is bad really*. *ED should be labelled like cigarettes " (FG5F3)*	-
		*"Age allowance for consumption should be regulated" (FG4F7)*	
		*"The vendor in the shop should ask about the age of the client before selling him an ED"(FG1M1)*	

*Proportion of respondents reporting similar input in the quantitative survey to the recurrent themes and subthemes emerging from the focus group discussions.

#### Individual-level factors

FGD participants reported a number of individual factors that affected their ED consumption behavior, including perceived benefits from consumption, experienced side effects (that did not affect their consumption behavior), and misinformation about content of ED. Survey respondents echoed several of these themes and subthemes as presented in Table 3.

*Perceived benefits of ED*. Survey results showed that the most important reasons behind the consumption of ED were as follows: getting energy to study (47%), to work (36%), to play sports (35%), and to prevent sleepiness (20%). In addition, greater than one quarter of ED consumers reported drinking these beverages during exam times rather than weekends and semester breaks. A more frequent consumption of ED was also reported during the fall and spring semesters compared to the summer semester (51.1% versus 31.3%, respectively). FGD participants reinforced the same reasons for consuming ED to get energy and stay awake, especially during exams (see Table 3). Previously, Itany *et al*. [8] showed that 78.5% of Lebanese children and university students in their pilot study perceived ED to have ‘energizing’ effects and to ‘stimulate wakefulness’. Alarmingly, a greater association was also found in the same study between the consumption of these highly caffeinated beverages during exam period and the adverse health effects reported by participants. Nevertheless, these effects didn’t deter the consumers’ drinking behavior [8]. It is worth noting that ED are highly marketed among young consumers and athletes for enhancing their physical performance and boosting their stamina [43], which can explain why our FGD participants and adolescents in other qualitative studies consume these beverages in an attempt to enhance their athletic performance [10, 27].

*Experienced side effects (did not affect ED consumption)*. When asked about potential side effects from ED consumption, 51.5% of survey respondents reported headache as one of the most frequently endured effects, after consuming ED alone or mixed with alcohol. In addition, a variety of mild to moderate symptoms were noticed by ED consumers within the present survey, such as increased heartbeat (42%), jitteriness/tremor/shakiness (36%), dizziness (30%), anxiety/nervousness/irritability (29%), fatigue (27%), and insomnia (19%). Nausea and vomiting (15%) after consuming ED and AmED were also reported mainly by female respondents. Some participants indicated being involved in ‘risky’ behaviors after consuming ED, such as getting into a verbal or physical arguments and fights (13%) and not using a condom in sexual practices (10%). Most notably, 12% of ED consumers reported that they required medical treatment at least once after consuming these beverages. FGD participants also reported experiencing several adverse side effects, including shakiness, dizziness, and increased heartbeat. One of the female participants stated: *“I drank it once while I had exams*, *I couldn't write*, *the pen was shaking in my hand*, *all my body was shaking*. *My heart was beating fast and I had no control on my extremities*. *It was so awkward it was caused by a one- time consumption but maybe I drank 3 cans”* (FG4F8). However, these side effects did not seem to affect the ED consumption behavior of university students, as several attributed these effects to the intake of AmED, consecutive drinking of coffee and EDs, drinking and smoking, or the subject's overall health status: *“Not because of the ED*, *maybe I drank a lot of coffee with it and I had tachycardia and my hand started shaking"* (FG1M1);*“Alcoholic ED do have side effects*, *many of my friends had some problems but the regular one [without alcohol]*, *it may only cause stress"* (FG1M6).

Similar to our results, 52% of study participants of a cross-sectional study on university students in UAE found that headache was the major experienced side effect following the consumption of EDs and AmED [24]. Other side effects of EDs and AmED that were reported in the literature included increased cardiovascular and blood pressure complications [16, 53]. The European Food Safety Authority and other studies stated that although current research is insufficient to draw conclusions about the safety of ED consumption, we cannot undermine the life-threatening risks of consuming excessive amounts of EDs or AmED, particularly in combination with physical exercise [54].

*Misinformation about content of EDs*. The lack of adequate knowledge with respect to the EDs ingredients was not identified within the quantitative survey, however, it was noted as a common subtheme that emerged from the FGD. Caffeine and sugar were the most recognized ingredients of ED; yet, some differences in the knowledge were noted between males and females. Male participants stated that "caffeine", "taurine", and "ginseng" were the main ingredients of ED, whereas females identified sugar as the only key ingredient of ED. It is worth noting that only a few participants reported ever trying to read ingredients or labels on ED cans, and some mentioned unrecognized ingredients such as "mice's urine", "bulls' sperm" and "testosterone”, which further reflected the misinformation regarding the content of ED. Those who did give accurate and detailed information about the content of ED were mostly students in health-related majors, including pre-med, pharmacy, and biology. In the study by Itany *et al*. [8], participants were asked to select ingredients of ED out of a given list of different substances, and caffeine and sugar were the most correctly marked components of ED by the study participants.

#### Interpersonal-social factors

Two subthemes emerged under the interpersonal factors theme that had an influence on the drinking behavior of focus groups participants: peer pressure & social image as well as parental influence.

*Peer pressure & social image*. Throughout the FGD, male and female participants appeared to be equally influenced by their peers when it comes to the use of ED. They expressed being encouraged and sometimes pressured to drink EDs when hanging out with their peers: “*If I’m in a group and I don’t want to drink*, *I feel pressured*, *they tell me like “Oh*, *you don’t drink*?!*”* (FG4F3); “*If you have a gathering for fun at your place*, *you cannot serve tea or orange juice*. *To go with flow*, *you need to have ED*. *More than 70% of our community like ED"* (FG6F8). In addition, some participants mentioned that they were more exposed to ED during college years as compared to their high school: *"at university you hear more about it*, *in school there wasn't too much exposure"* (FG5F1). All females in the focus groups had a common opinion that male consumers falsely perceive themselves as more "powerful", "cool", or "special" just by holding the ED can. Such a perception was indeed echoed by some male participants "*It's effective to people…I mean the view is effective*, *when people see you drinking*, *it's attractive*" (FG1M).

Similar observations were noted in the literature whereby youth and young adults reported trying ED in the company of their peers and associated this type of beverages with being ‘cool’ and attractive [14]. For example, a qualitative study by Visram and colleagues showed that ED consumption was linked to the desire of feeling attractive to the opposite sex, as well as to the shaping of self-identity [10]. Another study conducted amongst adolescents and young adults (16–35 years old) in Auckland, New Zealand showed that most of ED consumers tried their first drink with the company of a friend. In addition, peer influence was more pronounced amongst the youngest groups (16–21 years old) compared to the older groups who had more conscious knowledge of the detrimental health effects of these beverages [26]. Jones and colleagues further highlighted the symbolic attractiveness of AmED amongst consumers, who expressed that “being seen drinking [these beverages] might signify to others that they were popular or "cool" and that they fit within the "in-group" [16].

In terms of the social image, several participants stated that drinking ED and the choice of brand gives an indication of someone’s social status and the impression he wants to make: *“People who drink ED are like living in their own world*. *They’re like I drink [ED brand]*, *I’m a VIP”* (FG4F); “*First I look to what the person is drinking*. *Then I think you can assume the socioeconomic level of that person from what he drinks*. *For example*, *if he’s drinking [ED brand 1]*, *he has a limited income*. *If he has [ED brand 2]*, *then he comes from a higher class*” (FG1M4). Although the international ED and AmED brands available in Lebanon, such as Red Bull and Vodka Red Bull, were more expensive than the local ED products, our FGD participants still favored the foreign brands and perceived them as more credible and of higher quality. Preferences for international brands has been reported previously among consumers in developing countries [55], including those in Arab countries [56]. In addition, previous studies conducted among school and university students in Lebanon corroborated our results whereby participants reported Red Bull as their preferred brand [8, 38]. This brings forward the impact of aggressive marketing and branding strategies on consumers’ preference and loyalty to certain brands, regardless of their socioeconomic background, class, or purchasing power.

*Parental influence*. University students provided mixed evidence with regards to the role of parents and its influence on the ED consumption behavior of their children. Most of the FGD participants reported that parents do not support their children’s drinking behavior and participants reported that they avoided drinking in their parents’ presence. However, parental opposition did not stop study focus groups participants from consuming ED: *"I can't tell my parents that I drink [energy drink brand]*. *It's not that I do it in secret but I'd rather not hear lectures and stories*, *so I just drink it when I'm outside*" (FG6F7); "*Mom asked me to stop it (ED) but when I tell her that it helps me focus on my studies then she gets convinced"* (FG3M1). Nevertheless, a few male participants reported that their parents, in fact, consume ED and that helps in reinforcing their drinking behavior: *“My dad drinks [ED brand] all the time*, *more than I do*, *my mom wants us to stop*, *she never drinks" (FG2M2)*.

It is worth noting that the influence of parents on the drinking behavior of university students was not reported in the survey, yet it was raised in the FGD. Although the literature supports the role of parents as a powerful social factor affecting their children's health behaviors [57–59], this evidence is less consistent among young adults. For example, in a study exploring children and young adults perceptions on ED, some parents were misinformed about the content and effect of ED thus were indirectly encouraging their children drinking behavior while others didn't know about their children consumption [10]. However, a study exploring the association of social-environmental and individual-level factors with adolescent soft drink consumption among Dutch adolescents showed that parents' strict practices in regards to soft drinks consumption actually decreased adolescents' intake of the beverages and was moderated by the adolescent personality [48]. On the other hand, a study investigating the perceptions and patterns of ED use among Australian young adults showed that, similar to our findings, participants’ parental opposition to ED consumption was less likely to reduce or stop the consumption of their children [60]. It is worth noting that although the literature is not yet consistent on whether parental opposition can influence the consumption patterns of children, the involvement of parents in a specific eating or drinking behavior is rather conducive for the adoption of similar behaviors by children [61, 62].

#### Environmental factors

*S*everal environmental influences were identified as subthemes that affect the ED drinking behavior of focus groups participants. These subthemes included advertisements & branding, affordability & availability, and lack of policies & regulations.

*Advertisement & branding*. Appealing advertisements, strong branding, and reputation appeared to be key environmental stimuli for ED consumption among FGD participants. One of the key influences on participants' ED consumption was advertisements across various media types, which affected their choice for purchasing and consuming ED. Participants easily recalled the slogans and campaigns of ED and described them as "different", "strange", "smart", and "catchy". Males were mostly attracted by ads involving athletes or extreme sports while funny characters, songs, and entertainments caught females' attention: "*I really like them [ED]*, *one of the reasons for me to like a [specific brand] is the advertisement*. *What I like is that they make you believe that you can do better just by drinking it*. *I don't personally believe it but the ads are nice*" *(FG3M1); "The most thing I like about ED ads is seeing my preferred celebrity in it" (FG4F7)*.

Advertising and marketing were identified as powerful environmental factors affecting alcohol use and abuse in the United States [63]. In general, higher levels of exposure to commercial television and non-broadcast types of food marketing are associated with a greater consumption of advertised foods and beverages, particularly among young consumers [64]. In the case of ED and AmED, studies have shown that ED companies are using highly effective strategies to reach their consumers by involving their products into sporting events, sponsoring parties, advertising their drinks in connection with celebrities, or distributing these beverages for free in universities and public events [65].

*Affordability & availability*. Most of FGD participants considered that prices of ED are quite affordable and comparable to the prices of juices and soft drinks commonly consumed among young adults. In addition, they emphasized that the general availability of ED throughout the Lebanese market increased the ease of access and consumption of these beverages: *"If I don't find ED in my fridge*, *I get it from the grocery shop in the building"*, (FG5F1).

Similar to our findings, affordability and availability of ED and AmED were found as facilitators to consumption by adolescents in UK and Australia [10, 12]. These findings highlight the need for further studies to examine if introducing different taxing strategies to ED in Lebanon can be effective in reducing the drinking behavior of university students. These studies can be based on growing evidence in the field of public health showing that taxation of alcohol or sugar-sweetened beverages can reduce the intake of these beverages and contribute to improved health outcomes [66, 67]. However, it is still important to consider the effects of different ED taxing strategies on the consumption of other substitutes, such as sugar-sweetened and caffeine-rich beverages.

*Lack of policies & regulations*. Other environmental facilitators for ED consumption that were discussed by FGD participants included the *lack of policies* that regulate the sales and marketing of ED in Lebanon: *"I used to work in a place where most of alcoholic cocktails contained ED*. *We don't usually tell the clients that the beverages have ED" (FG4F*8).

When asked if they know of any regulations regarding ED in Lebanon, only few participants reported hearing that AmED may be banned and shared their agreement on the importance of regulating the sale of both ED and AmED. Participants suggested several options for regulating the sales of these beverages, including setting an age limit as a criteria for consumption, limiting the availability of AmED to bars and nightclubs, as well as controlling the content and timing of ED, and AmED advertisements on TVs or other media channels: *“They [government] should specify a legal age for ED consumption and give a notice to the sellers" (FG2M1)*; *"It annoys me when they use in their ads small cartoons characters that are appealing" (FG5F1)*.

Similar strategies to those suggested by our focus groups participants were reported earlier by a qualitative study conducted by Francis *et al*. (2017) when exploring the viewpoints of university students in Australia on how to decrease ED consumption among youth. Among the identified strategies were policies that place restrictions on ED consumption, such as banning the sale of large cans of ED as well as banning the sale of ED in schools and universities in addition to spreading awareness about ED’s health effects [68]. It is also worth noting that at the time of the data collection, a decree to ban the import and manufacturing of premixed AmED was being proposed by governmental authorities in Lebanon, including the Lebanese Ministry of Economy and Trade and the Ministry of Health. This decree aimed at banning the import, manufacturing, selling, and marketing of pre-mixed AmED, however, consumers could still order alcohol to be mixed with energy drinks in public bars and restaurants or prepare these drinks within their homes. The implementation of the decision to ban the import of AmED and prevent their marketing under penalty of legal action was implemented starting summer 2014 [69], however, questions remain to date as to the level of enforcement of this decree and to what extent are authorities imposing financial and legal repercussions on those who violate this law. Other regulatory measures are still lacking in the country including the lack of policies that set age limits for the purchase and consumption of ED and AmED, as well as insufficient regulations pertinent to the packaging and labeling of these beverages and their high caffeine content that can pose serious harm to consumers.

### Strengths and limitations

One of the strengths of the present study is the use of a mixed methods approach to explore quantitatively the consumption patterns and correlates of ED consumption followed by an in-depth investigation of the perceptions and experiences of college students towards this prevalent drinking behavior. To our knowledge, only one study to date used a similar mixed methods approach to explore this drinking behavior, however, researchers were focused on AmED only and the study was confined to children and youth (aged 12–17 years old) in Australia [12]. Another strength of the study is the alignment of the emerging themes with the social-ecological framework, which not only contributes to the understanding of multiple levels of influence of the health behavior in question but can also better guide public health practitioners in the development of successful interventions [70]. Nevertheless, the study has a number of limitations worth considering. The study was cross-sectional in nature limiting our ability to study any cause-effect relationships between ED consumption and the reported side effects. In addition, the study was conducted in a confined geographical location within Lebanon (Beirut capital) and using a convenience sample of university students from only two private universities, which limits the generalizability of the study findings to young adults across the country. Nevertheless, the research team made every attempt to reduce selection bias by recruiting university students from different socioeconomic backgrounds, disciplines, and locations across campuses, while also ensuring that both genders are well-represented in both the phases of the study. Another potential limitation for the study was social desirability, which may have deterred participants from reporting their high ED consumption or the potential effects that they have experienced. To overcome this challenge, the research team received adequate training to collect data using both the quantitative survey and qualitative FGD methods, while also using probing techniques that can assist respondents to reflect on their viewpoints and perceptions towards ED and AmED consumption referring to both their experiences and those of their siblings, peers, or others in their social circles.

## Conclusion

Findings from this mixed method study showed ED consumption is a common drinking behavior amongst university students in an urban setting in Lebanon, particularly among males. Participants in the quantitative and qualitative phases of the study reported several adverse health effects after consuming ED and AmED, such as increased heart rate, dizziness, and shakiness; however, these side effects did not seem to affect their consumption behaviors. Key themes that emerged from the focus groups highlighted multi-levels factors affecting the consumption of ED, as per the socioecological model, namely individual, interpersonal/social, and environmental influences. Our results highlight the need for evidence-based, tailored public health interventions worth considering to curb the growing public health phenomenon of ED consumption amongst university students in Lebanon and similar contexts. These interventions need to focus on the different levels of influence that may affect the drinking behavior of university students and young adults, namely: individual, interpersonal/social, and environmental influences.

At the individual and interpersonal/social levels, educational interventions and awareness campaigns are required to correct misperceptions and highlight the physical and psychological harms of ED consumption. In addition, community-based programs that can tackle the home environment and social networks of young consumers and young adults within their educational institutions can be mobilized as key components in modeling healthy drinking and lifestyle behaviors [71]. At the environmental level, policies are needed to impose clear regulations restricting the sales and marketing of ED beverages amongst young consumers. Other regulations related to the transparent and clear labelling of ED ingredients and their potential harms are needed together with setting evidence-based upper limits on caffeine consumption [15]. Furthermore, pricing and point of sale-related strategies can be considered to limit the affordability and availability of ED and AmED amongst consumers, including youth and young adults [52]. Finally, system-level interventions are needed to influence social and cultural norms and reduce ED acceptability and its appeal amongst young consumers.

## Supporting information

S1 Appendix Topic guide for focus group discussions with University Students. (DOCX)

S2 Appendix COnsolidated criteria for REporting Qualitative research (COREQ) checklist. (DOCX)
