# Supplementary material for: Energy drinks consumption and perceptions among University Students in Beirut, Lebanon: A mixed methods approach
Source: PLoS One. 2020 Apr 30;15(4):e0232199. doi: 10.1371/journal.pone.0232199 (PMC7192412; doi:10.1371/journal.pone.0232199)
Supplement: S1 Appendix — (DOCX) [file pone.0232199.s001.docx]

**S1. Topic Guide for Focus Group Discussions with University Students**

# Introduction

Hello everyone, thank you for participating in our focus group study.

My name is X, I'm a graduate student at AUB and the focus group session we are having today is part of my thesis project. My colleague X will also be attending this session and will be taking notes of our discussion.

The reason we are having this focus group today is to mainly find out what are the perceptions and attitudes of young university students like you towards energy drinks. Thus, you are gathered hereto share your experience, thoughts and opinions aboutthese beverages.There are no wrong or right answers so I would like to hear everyone's point of view and hope you canbe honest and express yourself with no hesitation even when your responses may not be in agreement with the rest of thegroup.

First of all, let me remind you that this session will be audio recorded only to help me remember all the information we will be sharing and no one besides the AUB research team will be allowed to access the recordings. No names or other identifiers will be attached to your comments and all your responses will be kept anonymous. Although it might be tempting for you to share what you've heard with your friends and family, I wish to bring your attention to the importance of keeping the individual responses confidential. This will make you all feel comfortable sharing your thoughts. This focus group session will take 60-90 minutes.

# Focus group script

You are all gathered in this group today because you share one common characteristic which is "energy drink consumption".

Icebreaker question: As you have noticed, we have placed a selection of beverages in the center, is everybody familiar with these beverages? These beverages are from the energy drinks (ED) family.

1. What do EDs mean to you? *(Probing questions: How do you define EDs? Do you know what the ingredients of ED are?)*
2. Can you tell us more about how did you learn about EDs? Who or what encouraged you to try EDs the first time you tried them?

*(Probing questions: a-* When (at what age) did you first try EDs? b- *Did you first try them alone, with parents, family, friends, others? c- Was your first trial related to/ triggered by a special event?)*

1. What made you repurchase EDs after the first time you consumed them? What are the main reasons for consuming these beverages?
2. Do you regularly consume EDs? When you purchase EDs, what are the factors you consider the most important to you?
3. How do EDs influence you? *(In your opinion, are there any benefits to EDs? Can you provide us with examples of these benefits that you may have experienced?)*
4. Have you heard about or experienced yourself any side effects while consuming EDs or after consuming alcohol mixed with EDs (AmEDs)? Can you share with us some of these side effects?
5. Which brand(s) of EDs do you consume most? And what are the reasons for your choice?
6. What do you think about ED advertisements (ads)? (Probing questions: a- What are the most powerful/ expressive ads that catch your attention? b- *What do you mostly like/dislike about these ads?)*
7. How do you describe people who drink EDs?

*(Probing questions: a- What do you think about people who consume these beverages? , b- Do you describe or perceive those who consume EDs as any of these options? Cool, brave, sportive, social? Other? , c- If you are with a group of friends drinking ED except one who refuses to, what will you probably think about this person?)*

1. In your opinion, how do others think about you when they know you consume EDs or AmEDs? *(Probing questions: what do your parents think about the fact that you consume EDs? What do your friends think? Others?)*

1. Have you ever considered or are currently considering reducing your intake or stopping EDs consumption? If so, why? What factors, if any, may influence your choice to stop drinking EDs?
2. Do you keep EDs cans/bottles in your fridge or buy them only when needed? Why?
   1. What would you do if you felt like having EDs when for some reasons they are not accessible?

*(If you wanted an ED while you are in a restaurant, shop, pub, any place where EDs are not available, what will you do?)*

1. Have you heard about any regulations concerning energy drinks in Lebanon? Do you think that the sales or marketing of energy drinks need to be regulated by the government as in other countries? Please explain.
2. Please feel free to share with us any other thoughts or opinions that you would like to add as we wrap up our discussion.

Thank you for your time!
